# Modulation of Leptin and Leptin Receptor Expression in Mice Acutely Infected with *Neospora caninum*

**DOI:** 10.3390/pathogens9070587

**Published:** 2020-07-17

**Authors:** Luzia Teixeira, Alexandra Correia, Bárbara M. Oliveira, Ana Pinto, Paula G. Ferreira, Manuel Vilanova

**Affiliations:** 1 ICBAS—Instituto de Ciências Biomédicas Abel Salazar, Universidade do Porto, Rua de Jorge Viterbo Ferreira, 228, 4050-313 Porto, Portugal; alexandra.correia@ibmc.up.pt (A.C.); bmnoliveira@icbas.up.pt (B.M.O.); arpinto@icbas.up.pt (A.P.); pferreir@icbas.up.pt (P.G.F.); vilanova@icbas.up.pt (M.V.); 2 UMIB—Unidade Multidisciplinar de Investigação Biomédica, Universidade do Porto, Rua de Jorge Viterbo Ferreira, 228, 4050-313 Porto, Portugal; 3 i3S-Instituto de Investigação e Inovação em Saúde, Universidade do Porto, Rua Alfredo Allen, 208, 4200-135 Porto, Portugal; 4 IBMC—Instituto de Biologia Molecular e Celular, Universidade do Porto, Rua Alfredo Allen, 208, 4200-135 Porto, Portugal

**Keywords:** *Neospora caninum*, leptin, leptin receptor

## Abstract

*Neospora caninum* is an apicomplexan parasite that in cattle assumes particular importance, as it is responsible for abortions reported worldwide. Leptin is an adipokine mainly secreted by adipocytes, which beside its role in maintaining metabolic homeostasis also has important effects in both innate and adaptive immunity. In previous work, we showed that mice chronically infected with *N. caninum* had elevated serum leptin levels. Here, we sought to assess whether acute infection with *N. caninum* infection influenced the production of this adipokine as well as leptin receptor mRNA levels. Our results show that acute infection with *N. caninum* led to decreased leptin serum levels and mRNA expression in adipose tissue. A decrease in leptin receptor transcript variant 1 mRNA (long isoform) and leptin receptor transcript variant 3 mRNA (one of the short isoforms) expression was also observed. An increase in the number of cells staining positive for leptin in the liver of infected mice was observed, although this increase was less marked in Interleukin (IL)-12/IL-23 p40-deficient mice. Overall, our results show that *N. caninum* infection also influences leptin production during acute infection.

## 1. Introduction

*Neospora caninum* is an apicomplexan parasite, phylogenetically close to *Toxoplasma gondii*, that was first described as a causative agent of neurologic disease in dogs [1,2]. Although *N. caninum* has been isolated from several animal species, neosporosis assumes particular importance in cattle, where it is responsible for abortions reported in many countries [1,3]. Bovine neosporosis leads to heavy economic losses in the dairy and beef industry, and no vaccine exists to prevent this infection [3,4]. Resistance against neosporosis has been associated with host IL-12 and IFN-γ production. Mice genetically deficient for these cytokines [5,6] or mice in which these cytokines were neutralized with specific monoclonal antibodies [7] were shown to be lethally susceptible to *N. caninum* infection. In the murine model, our latest work uncovered noticeable immune cell alterations occurring in the adipose tissue during *N. caninum* infection that persisted long after local parasite elimination [8,9]. Early on in infection, distinct lymphoid cell populations, such as CD4^+^ and CD8^+^ TCRβ^+^ cells, TCRγδ^+^ cells and NK and NK T cells, were shown to contribute to IFN-γ production in both visceral and subcutaneous adipose tissue [8]. This production of IFN-γ was largely abrogated in the lethally susceptible IL-12/IL-23 p40-deficient mice [8]. In these mice, the adipose tissue is heavily colonized by *N. caninum* seven days after infection, as seen by immunohistochemistry analysis, contrastingly to wild-type mice where parasites are rarely detected by the same technique at this time point [9]. The contribution of the adipose tissue to the immune response can occur through the immune cells resident in this tissue or due to the influence of adipokines produced by adipocytes in cells of the immune system [10]. Leptin is an adipokine mainly secreted by adipocytes, which beside its role in maintaining metabolic homeostasis [11] also has important effects in both innate and adaptive immunity [12,13]. For example, leptin promotes Th1 responses [14] and is essential for effector T cell activation [15]. Indeed, many works have shown immune cells have leptin receptors and are influenced by this adipokine [16] (reviewed in [13,17]). In mice, at least six isoforms of the leptin receptor were described [18]. The long isoform (isoform b, known as Ob-Rb or LepRb) is the main isoform responsible for leptin signalling, capable of activating for example the JAK (Janus kinase)–STAT (signal transducers and activators of transcription) signalling pathway among others [18,19]. Nevertheless, activation of the short isoform a (known as Ob-Ra or LepRa) can lead in some extent to MAPK (mitogen-activated protein kinase) activation [20]. Other studies have also suggested that ObRa can also be involved in leptin transport [21]. In previous work, we showed that mice chronically infected with *N. caninum* had elevated serum leptin levels [9]. In this work, we wanted to assess whether during acute infection the production of leptin was also affected, as well as the levels of leptin receptor mRNA of two major transcript variants, the leptin receptor (*Lepr*) transcript variant 1 mRNA (also known as leptin receptor variant b) and leptin receptor (*Lepr*) transcript variant 3, mRNA (also known as leptin receptor variant a). These parameters were evaluated not only in wild-type C57BL/6 mice but also in IL-12/IL-23 p40-deficient mice which, contrarily to wild-type (WT) mice, are lethally susceptible to *N. caninum* infection [6]. Our results show that acute infection with *N. caninum* led to a decrease in the levels of leptin that was also accompanied by decreased leptin receptor expression in adipose tissue. On the other hand, an increase in the number of cells staining positive for leptin was observed in the liver of wild-type infected mice, contrastingly with the slight increase observed in IL-12/IL-23 p40-deficient mice. Altogether, these results hint that *N. caninum* infection modulates leptin production and signalling.

## 2. Results

### 2.1. Leptin Levels in the Serum of *N. caninum*-Infected Mice

**Figure 1:**
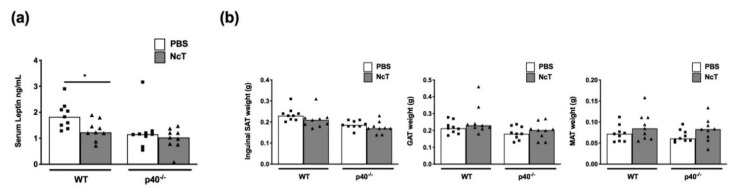
Decreased leptin serum levels in mice acutely infected with *Neospora caninum* is not associated with adipose tissue weight change. (**a**) Leptin serum levels and (**b**) weight of inguinal subcutaneous adipose tissue (SAT), gonadal adipose tissue (GAT), and mesenteric adipose tissue (MAT) weight, as indicated, of wild-type (WT) and IL-12/IL-23 p40-deficient (p40^−/−^) mice 24 h after administration of 1 × 10^7^ *N. caninum* tachyzoites (NcT) or phosphate-buffered saline (PBS), intraperitoneally. Bars represent the median values of nine mice per group, with each individual mouse being represented by a symbol. These are pooled results from three independent experiments with three mice per group, per experiment. Statistically significant differences between *N. caninum*-challenged and respective control groups are indicated (Mann–Whitney test, * *p* < 0.05).

In a previous work, we observed increased serum levels of leptin 21 days and 2 months after infection in wild-type C57BL/6 mice [9]. In this work, we intended to determine whether the levels of this adipokine could be affected at an early time point after infection in wild-type C57BL/6 mice (WT) and in IL-12/IL-23 p40-deficient (p40^−/−^) mice, which contrarily to WT mice, are lethally susceptible to *N. caninum* infection [6]. As shown in Figure 1a, a slight decrease of leptin serum levels was detected 24 h after infection in WT mice, while no alteration was observed in p40^−/−^ mice. No differences in the weight of inguinal subcutaneous adipose tissue (SAT) (as surrogate marker of subcutaneous adipose tissue), gonadal adipose tissue (GAT), and mesenteric adipose tissue (MAT) were observed between infected and phosphate-buffered saline (PBS) control groups (Figure 1b).

### 2.2. Leptin mRNA Expression Levels in Adipose Tissue of *N. caninum*-Infected Mice

**Figure 2:**
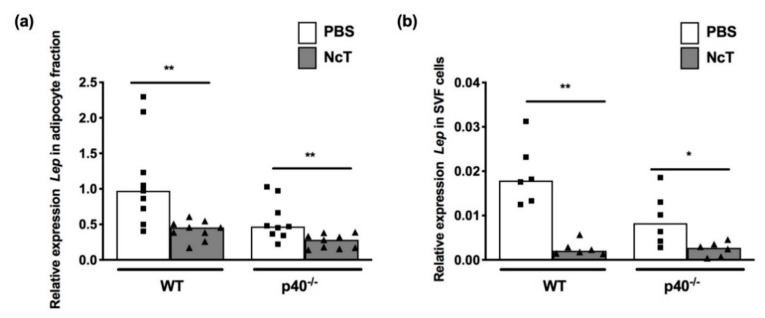
Decreased expression of leptin in the adipose tissue of *Neospora caninum*-infected mice. Relative levels of Leptin (*Lep*) mRNA normalized to non-POU-domain containing octamer binding protein (*Nono*) mRNA, detected by real-time PCR in the (**a**) adipocyte fraction or (**b**) stromal vascular fraction (SVF) cells of mesenteric adipose tissue of wild-type (WT) and IL-12/IL-23 p40-deficient (p40^−/−^) mice 24 h after intraperitoneal administration of 1 × 10^7^ *N. caninum* tachyzoites (NcT) or PBS. Bars represent the median values of (**a**) nine mice or (**b**) six mice per group, with each individual mouse being represented by a symbol. Results are pooled from (**a**) three independent experiments or (**b**) two independent experiments, with three mice per group, per experiment. Statistically significant differences between *N. caninum*-challenged and respective control groups are indicated (Mann–Whitney test, * *p* < 0.05; ** *p* ≤ 0.01).

We next assessed leptin mRNA expression levels in adipose tissue, as it is the main source of serum leptin [22]. The mesenteric adipose tissue was separated into two fractions after collagenase digestion and centrifugation, the adipocyte fraction (floating layer) and the stromal vascular fraction (SVF) (cells at the bottom of the tube as a pellet). Accordingly with leptin being produced mainly by adipocytes in the adipose tissue, leptin mRNA levels in the adipocyte fraction of control mice were approximately 60 times higher than the leptin mRNA levels in the SVF cells (Figure 2a,b, median value WT PBS adipocytes = 0.9727 vs. 0.01789 for WT SVF cells when normalized to non-POU-domain containing octamer binding protein (*Nono)* mRNA, *p* = 0.0004 with Mann–Whitney test; median value p40^−/−^ PBS adipocytes = 0.4698 vs. 0.008278 for p40^−/−^ SVF cells when normalized to *Nono* mRNA, *p* = 0.0004 with Mann–Whitney test). A decrease in leptin mRNA expression levels in the adipocyte fraction was detected in both WT and p40^−/−^ mice infected with *N. caninum* compared to non-infected counterparts (Figure 2a and Supplementary Figure S1). In the SVF, a decrease in leptin mRNA levels was also observed upon *N. caninum* infection of both WT and p40^−/−^ mice (Figure 2b and Supplementary Figure S1).

### 2.3. Leptin Receptor mRNA Expression Levels in Adipose Tissue of *N. caninum*-Infected Mice

**Figure 3:**
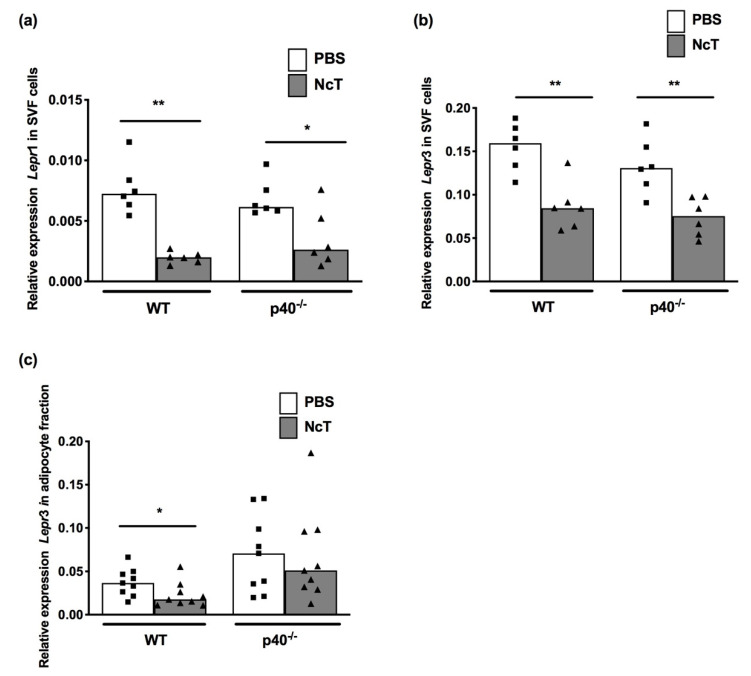
Decreased expression of leptin receptor in the adipose tissue of *Neospora caninum*-infected mice. (**a**) Relative levels of leptin receptor transcript variant 1 (*Lepr*1) mRNA and (**b**) leptin receptor transcript variant 3 (*Lepr*3) mRNA, normalized to non-POU-domain containing octamer binding protein (*Nono*) mRNA, in stromal vascular fraction (SVF) cells of mesenteric adipose tissue of wild-type (WT) and IL-12/IL-23 p40-deficient (p40^−/−^) mice 24 h after intraperitoneal administration of 1 × 10^7^ *N. caninum* tachyzoites (NcT) or PBS, as indicated. Bars represent the median values of six mice per group, with each individual mouse being represented by a symbol. These are pooled results from two independent experiments with three mice per group, per experiment. (**c**) Relative levels of leptin receptor transcript variant 3 (*Lepr*3) mRNA, normalized to non-POU-domain containing octamer binding protein (*Nono*) mRNA, detected by real-time PCR in adipocyte fraction of mesenteric adipose tissue of wild-type (WT) and IL-12/IL-23 p40^−/−^ (p40^−/−^) mice 24 h after intraperitoneal administration of 1 × 10^7^ *N. caninum* tachyzoites (NcT) or PBS, as indicated. Bars represent the median values of nine mice per group, with each individual mouse being represented by a symbol. These are pooled results from three independent experiments with three mice per group, per experiment. Statistically significant differences between *N. caninum*-challenged and respective control groups are indicated (Mann–Whitney test, * *p* < 0.05; ** *p* ≤ 0.01).

Having determined that *N. caninum* infection influenced leptin mRNA levels, we assessed whether transcription of the leptin receptor could also be influenced by infection. We assessed the long isoform, leptin receptor transcript variant 1 (*Lepr*1) mRNA, and one of the short isoforms, leptin receptor transcript variant 3 (*Lepr*3) mRNA. In adipocytes, no specific product was observed for *Lepr*1 mRNA. Contrastingly, in the SVF *Lepr*1 mRNA was detected and the levels were decreased upon infection in WT mice (Figure 3a). In p40^−/−^ mice, *Lepr*1 expression mRNA levels were only observed to be decreased upon infection when the data were normalized to *Nono* mRNA levels, and no change was observed when they were normalized to *Hprt* mRNA levels (Figure 3a and Supplementary Figure S2a). *Lepr*3 expression was detected in both adipocyte fraction and SVF cells, with its expression levels being higher in the latter than in the former (Figure 3b,c, median value WT PBS adipocytes = 0.03665 vs. 0.1594 for WT SVF cells, *p* = 0.0004 with Mann–Whitney test; median value p40^−/−^ PBS adipocytes = 0.07081 vs. 0.1308 for p40^−/−^ SVF cells, *p* = 0.0360 with Mann–Whitney test). In the SVF fraction, there was a reduction in the *Lepr*3 mRNA expression levels upon infection in both WT and p40^−/−^ mice (Figure 3b and Supplementary Figure S2b). Upon *N. caninum* infection, the mRNA levels of this receptor were slightly decreased in the adipocyte fraction of WT mice, and no difference was observed in p40^−/−^ mice (Figure 3c and Supplementary Figure S2c).

### 2.4. Leptin Detection by Immunohistochemistry

**Figure 4:**
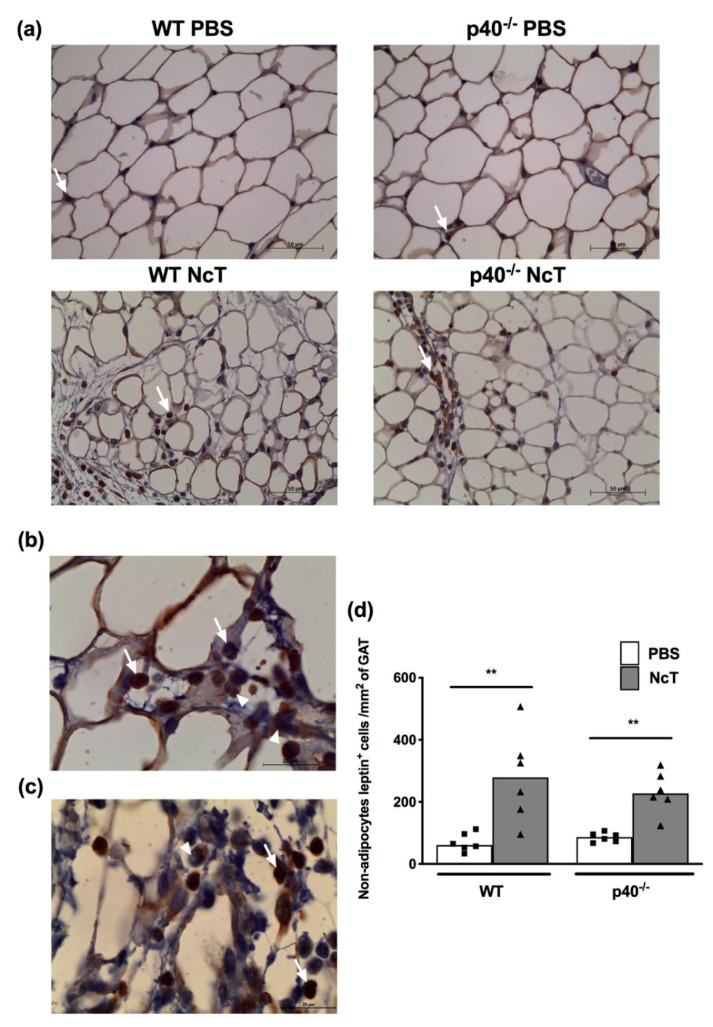
Staining for leptin in the adipose tissue of *Neospora caninum*-infected mice. (**a**) Representative immunohistochemistry analysis of leptin in gonadal adipose tissue (GAT) of wild-type (WT) and IL-12/IL-23 p40-deficient (p40^−/−^) mice 24 h after administration of 1 × 10^7^ *N. caninum* tachyzoites (NcT) or PBS. GAT was specifically stained (brown colouration) with a polyclonal anti-mouse leptin antibody and counterstained with haematoxylin. Arrows indicate examples of cells identified as non-adipocyte leptin^+^ cells. Bar = 50 μm in all micrographs. High magnification of staining for leptin in adipose tissue of (**b**) WT and (**c**) p40^−/−^ mice, 24 h after administration of 1 × 10^7^ NcT, showing cells with morphology compatible with polymorphonuclear cells (arrow) and mononuclear cells (arrowhead). Bar = 20 μm. (**d**) Number of non-adipocyte cells staining positively for leptin (leptin^+^) per mm^2^ of GAT 24 h after challenge, counted in several tissue sections as illustrated in **(a)**. Bars represent the median values of six mice per group, with each individual mouse being represented by a symbol. These are pooled results from two independent experiments with three mice per group, per experiment. Statistically significant differences between *N. caninum*-challenged and respective control groups are indicated (Mann–Whitney, ** *p* ≤ 0.01).

Leptin staining in adipose tissue was also assessed by immunohistochemistry in GAT. A widespread staining for leptin was observed not only in adipocyte cytoplasm but also in cells scattering the tissue (Figure 4a). In both WT and p40^−/−^ infected mice, beside adipocytes, staining was easily observed in both mononuclear and polymorphonuclear cells scattered in the tissue (Figure 4b,c). A higher number of non-adipocyte cells staining for leptin were observed in WT and p40^−/−^ infected mice when compared to the respective control mice group (Figure 4d). Illustrative examples of the micrographs where this analysis was made are shown in Figure 4a. The tissue is heterogeneous regarding leptin staining with aggregation of leptin^+^ cells in some areas (Supplementary Figure S3a). We have previously shown that parasites could be detected in gonadal adipose tissue as soon as 6 h after infection [9]. Accordingly, we detected parasites by immunohistochemistry in gonadal adipose tissue in both WT and p40^−/−^ mice (Supplementary Figure S4a).

**Figure 5:**
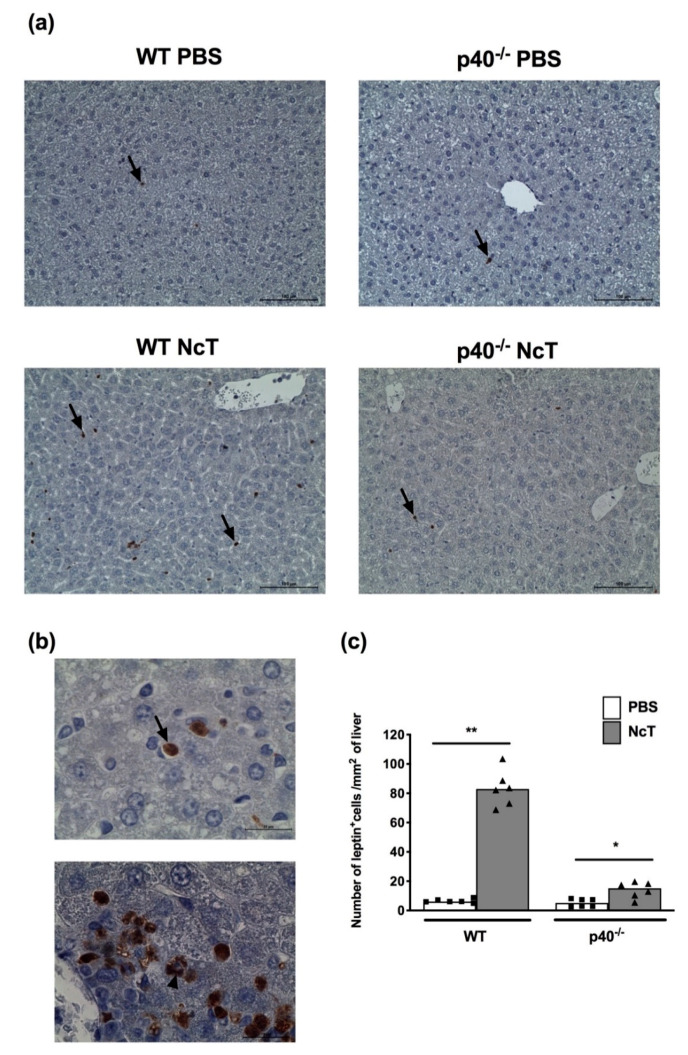
Increased number of cells staining for leptin in the liver of *Neospora caninum*-infected mice. (**a**) Representative immunohistochemistry analysis of leptin in the liver of wild-type (WT) and IL-12/IL-23 p40-deficient (p40^−/−^) mice 24 h after intraperitoneal administration of 1 × 10^7^ *N. caninum* tachyzoites (NcT) or PBS. Liver sections were specifically stained (brown colouration) with a polyclonal anti-mouse leptin antibody and counterstained with haematoxylin. Arrows indicate examples of cells identified as leptin^+^ cells Bar = 100 μm in all micrographs. (**b**) Cells with morphology compatible with polymorphonuclear cells (arrow) and mononuclear cells (arrowhead) are observed. Illustrative example of infected wild-type mice. Bar = 20 μm in all micrographs. (**c**) Number of cells staining for leptin (leptin^+^) per mm^2^ of liver 24 h after challenge, analysed in several tissue sections as illustrated in (**a**). Bars represent the median values of six mice per group, with each individual mouse being represented by a symbol. These are pooled results from two independent experiments with three mice per group, per experiment. Statistically significant differences between *N. caninum*-challenged and respective control groups are indicated (Mann–Whitney, * *p* < 0.05; ** *p* ≤ 0.01).

Since the liver is an organ already described to be colonized in acute infection by *N. caninum* [23], we also assessed leptin in this tissue using immunohistochemistry. Indeed, in the group of animals analysed we detected the parasite in the liver by immunohistochemistry, although scarcely, in all infected mice (Supplementary Figure S4b). In non-infected mice, leptin staining was seldom observed and was mainly confined to non-hepatocyte cells (Figure 5a). Upon infection with *N. caninum*, an increase in the number of non-hepatocytes cells staining for leptin was observed (Figure 5a). Polymorphonuclear cells staining for leptin were observed in both non-infected and infected WT and p40^−/−^ mice (Figure 5a,b). In the infected WT mice, a marked increase (12.8-fold increase) was observed in the number of cells staining for leptin. In p40^−/−^ mice, only a slight increase was observed (2.7-fold increase) in infected mice compared to non-infected mice (Figure 5c). Accordingly, non-hepatocyte cells were more easily observed in haematoxylin–eosin stained liver sections of infected WT mice compared to non-infected controls. In p40^−/−^ mice, differences between infected and control mice were not so evident, although, some areas of infiltration could also be observed (Supplementary Figure S5).

### 2.5. Leptin and Leptin Receptor mRNA Expression Levels in Liver of *N. caninum*-Infected Mice

**Figure 6:**
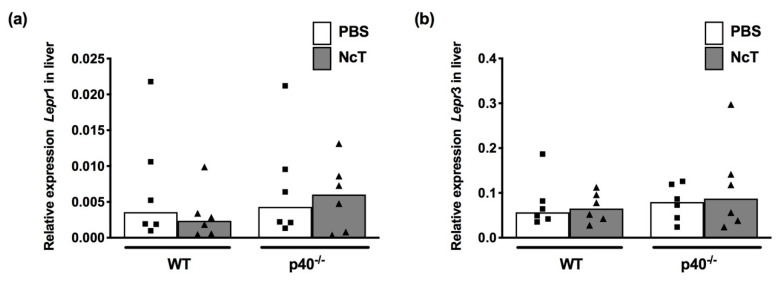
Expression of leptin receptor in the liver of *Neospora caninum*-infected mice. (**a**) Relative levels of leptin receptor transcript variant 1 (*Lepr*1) mRNA and (**b**) leptin receptor transcript variant 3 (*Lepr*3) mRNA, normalized to hypoxanthine guanine phosphoribosyl transferase (*Hprt*) mRNA, detected by real-time PCR in the liver of wild-type (WT) and IL-12/IL-23 p40-deficient (p40^−/−^) mice 24 h after intraperitoneal administration of 1 × 10^7^ *N. caninum* tachyzoites (NcT) or PBS, as indicated. Bars represent the median values of six mice per group, with each individual mouse being represented by a symbol. These are pooled results from two independent experiments with three mice per group, per experiment. Statistically significant differences between *N. caninum*-challenged mice and respective control groups were assessed by Mann–Whitney test.

As we detected increased number of leptin^+^ cells in the liver, we also assessed leptin and leptin receptor mRNA expression levels in this organ. Nevertheless, no leptin mRNA was detected in the whole liver (both in WT and p40^−/−^ mice infected and non-infected (n = 3 in each group, repeated in two independent experiments). Furthermore, no differences were found in *Lepr*1 and *Lepr*3 mRNA expression levels in the whole liver upon infection in either WT or p40^−/−^ mice (Figure 6a,b).

## 3. Discussion

We previously showed that mice infected for 21 days and 2 months with *N. caninum* presented increased levels of serum leptin [9]. Similarly, others have reported that rats infected with the closely related parasite *T. gondii* presented higher plasma leptin levels 4 weeks after infection [24]. Here, we found that earlier after infection, leptin levels were decreased in the serum of *N. caninum*-infected mice. It is known that the amount of circulating leptin is proportional to the amount of adipose tissue [18]. Since no differences were found in the amount of inguinal subcutaneous adipose tissue (used as a surrogate marker of subcutaneous adipose tissue), gonadal or mesenteric adipose tissue between control and infected mice, the decreased leptin concentration found in the latter are not likely due to different amounts of adipose tissue. This decrease in serum levels could, however, result from the observed decrease in leptin mRNA levels in the adipocyte fraction upon infection since adipocytes are the main source of circulating leptin [22]. Leptin mRNA expression was 60 times higher in the adipocyte fraction than in the stromal vascular fraction. This is in accordance with leptin mRNA being expressed by mature adipocytes and not by preadipocytes 3T3-L1 [25]. Cells that can be found in the stromal vascular fraction of adipose tissue, such as mast cells [26] and Treg [27], have been shown to express leptin mRNA [26,28]. Whether the reduction in leptin observed in the stromal vascular fraction could be due to reduced leptin mRNA expression in any of these cell populations remains to be determined in future studies. In accordance with detected leptin mRNA expression, adipocytes stained positive for leptin using immunohistochemistry, with a pattern similar to that observed by others [29,30], with staining visible in the cytoplasmic rim of the adipocyte. Cells scattered in the adipose tissue staining for leptin were also observed and were in higher number in both WT and p40^−/−^ infected mice. Since no upregulation was observed in leptin or leptin receptor mRNA levels in the stromal vascular fraction of infected mice, these cells may be recruited to the adipose tissue with leptin bound to the leptin receptor. Alternatively, the leptin-positive cells could have produced this adipokine but having already downregulated leptin mRNA.

Regarding leptin receptors, we were able to detect the long isoform Ob-Rb (*Lepr*1) only in the stromal vascular fraction and not in the adipocyte fraction. Others have already found low levels of Ob-Rb RNA in whole white adipose tissue [31]. The short isoform assessed in this work, Ob-Ra (*Lepr3*), was detected in both fractions with expression levels higher in the stromal vascular fraction cells than in adipocytes, which may reflect the fact that this short isoform has been associated with leptin transport [21]. Different types of cells can express different types of isoforms. The mRNA levels of both leptin receptor isoforms were decreased upon infection in the stromal vascular fraction of adipose tissue in wild-type mice. The consequences of this decrease are unknown in *N. caninum* infection. Nevertheless, in mice infected with the protozoan *Trypanosoma cruzi*, deficiency of leptin receptors is associated with increased parasitaemia and mortality rate [32].

Contrastingly to adipose tissue, no leptin mRNA expression was detected in the liver, although we did detect mRNA for leptin receptor transcript variant 1 and 3 and leptin protein by immunohistochemistry. Similarly, others did not detect leptin mRNA in the liver of adult male Wistar rats but detected leptin receptor mRNA and leptin staining by immunohistochemistry that was limited to non-hepatocytes cells [33]. We observed polymorphonuclear cells stained with leptin in both adipose tissue and liver. Using immunocytochemistry, others have previously reported that polymorphonuclear neutrophils stained for leptin [34] and leptin receptor as well [34,35]. The absence of leptin mRNA expression in our experiment in the liver could then be due to leptin mRNA expression levels below PCR detection level or it may indicate that exogenous-produced leptin is bound to leptin receptors in these cells. In contrast to the decreased levels of circulating leptin, we observed increased numbers of cells staining positive for leptin in the liver. This could result from uptake of leptin by activated cells, by binding to its receptor, which can lead to a decrease of systemic leptin levels. Others have shown that RAW-264.7 macrophages incubated for 24 h with Gram-negative bacteria *Salmonella typhimurium* increased leptin receptor expression and leptin uptake from the culture medium [36]. Interestingly, in this work, inhibition of leptin signalling increased the ability of macrophages to eliminate *S. typhimurium* [36]. Leptin-deficient mice infected with *Mycobacterium avium* infection presented lower splenic bacterial load over time and higher activation of T cells [37]. Dendritic cells from mice deficient in leptin also induced higher production of IFN-γ in mixed lymphocyte reactions and antigen-specific assays [37]. Therefore, it remains to be determined whether the early decrease in serum leptin detected upon *N. caninum* infection in WT mice could have also contributed to the higher frequency of IFN-γ producing cells observed in WT mice compared to p40^−/−^ mice 24 h after infection [8]. In other infection models, however, leptin has been attributed host-protective effects (reviewed in [17]). In *N. caninum* infection, the role of leptin and which cells are staining for leptin in both adipose tissue and liver remain to be determined. We have recently shown an association between *N. caninum* seropositivity and higher frequency of macrophages in bovine adipose tissue [38], which is in line with the increase in macrophage numbers observed in mice experimentally infected with *N. caninum* [9]. It would therefore be interesting to observe whether, in the bovine host, leptin and leptin receptor levels are also changed upon *N. caninum* infection. Reduced levels of leptin were observed in bovines experimentally infected with the nematode *Ostertagia ostertagi* without any change in weight gain [39]. In the lethally susceptible p40^−/−^ mice, no decrease in serum leptin was observed upon infection with *N. caninum,* but a decrease in leptin mRNA expression levels in the adipocyte fraction and stromal vascular fraction cells was observed, similar to the one observed in WT mice. This hints that mechanisms leading to leptin expression levels reduction are not greatly affected in the absence of the IL-12/IL-23 p40 subunit. In the liver, the increase of cells staining positive for leptin was less pronounced in p40^−/−^ mice that in wild-type mice. Whether this reflects lower recruitment of cells expressing leptin to the liver or lower binding of leptin to the cells remains to be determined in future studies. Overall, our work shows that *N. caninum* infection influences leptin production and leptin receptor expression early on in infection. How this affects the immune response of the host to infection remains to be determined in future studies.

## 4. Materials and Methods

### 4.1. Mice

Animals and housing conditions were previously described [8]. Briefly, WT female C57BL/6 mice (age between 7 and 8 weeks old) were acquired from Charles River and kept during the experiments at the animal facilities of Instituto de Ciências Biomédicas Abel Salazar (ICBAS) (Porto, Portugal) in ventilated cages. IL-12/IL-23 p40-deficient C57BL/6 mice were bought from Jackson Laboratories (Bar Harbor, ME, USA), bred and maintained at ICBAS also in ventilated cages. Hiding and nesting materials were provided. All procedures involving mice were executed following the European Convention for the Protection of Vertebrate Animals used for Experimental and Other Scientific Purposes (ETS 123) and directive 2010/63/EU of the European parliament and of the council of 22 September 2010 on the protection of the animals used for scientific purposes, as well as Portuguese rules (DL 113/2013). Authorization to perform the experiments was issued by competent national board authority, Direcção-Geral de Alimentação e Veterinária (0420/000/000/2012 and 0421/000/000/2015) and Institutional Ethical Committee. The majority of the samples analysed in this study were recovered at the time of sacrifice of animals described in another article [8], leading therefore to a great reduction in the sacrifice of new animals specifically for the research reported here.

### 4.2. Parasite Isolation and Challenge Infection

The parasite isolation was done as previously described in detail [40]. Briefly, *N. caninum* tachyzoites (NcT) of the Nc-1 isolate (ATCC^®^ 50843) were maintained by serial passage in VERO cells cultured at 37 °C, 5% CO_2_, in minimum essential medium with Earle’s salts (Sigma, St. Louis, MO, USA) supplemented with 2 mM L-glutamine, 200 units/mL of penicillin and 200 μg/mL of streptomycin (all from Sigma) and 10% foetal calf serum (Gibco: Invitrogen Corporation, Carlsbad, CA, USA). Tachyzoites were isolated from VERO cells by a column method previously described by others [41]. Briefly, VERO cells infected with *N. caninum* were cultured till the monolayer of cells was 70% destroyed. The adherent cells were harvested using a cell scraper together with the culture supernatants and all the content of the flasks was centrifuged at 1500× *g* for 15 min. The pellet was resuspended in phosphate-buffered saline (PBS), passed through a 25 gauge needle, and washed three times in PBS by centrifugation at 1500× *g* for 15 min. The final pellet was resuspended in 3 mL of PBS and passed through a PD-10 column filled with Sephadex G-25M (Amersham Biosciences Europe GmbH, Freiburg, Germany), previously equilibrated with PBS. The concentration of the parasite suspension was determined in a haemocytometer. The parasites used in our experiments underwent less than fifteen in vitro passages from the original vial, since others have shown attenuated virulence of *N. caninum* if maintained for a long time in tissue culture [42]. Viability of the *N. caninum* inocula was confirmed in the highly susceptible p40^−/−^ mice.

The challenge protocol was the one previously described [8]. Briefly, 8–20-week-old female WT or p40^−/−^ B6 mice were inoculated intraperitoneally (i.p.) with 1 × 10^7^ *N. caninum* tachyzoites diluted in 0.5 mL PBS (n = 3/group/experiment). Mock-infected controls were similarly i.p. injected with 0.5 mL of PBS (n = 3/group/experiment).

### 4.3. Collection of Biological Samples

Sample collection was done as previously described [8]. Briefly, twenty-four hours after infection, challenged mice were isoflurane anesthetized for retro-orbital blood collection and euthanized by cervical dislocation. Mesenteric adipose tissue (MAT, the visceral adipose tissue between the two peritoneal layers of the mesentery) was removed, its weight was measured, and the tissue was placed in Hanks’s balanced salt solution supplemented with 4% bovine serum albumin (BSA) and 10 mM HEPES buffer (all from Sigma-Aldrich) for further analysis. To minimize variability, all removed mesenteric adipose tissue was used for isolation of adipocyte fraction and stromal vascular fraction cells. Subcutaneous adipose tissue (SAT) and gonadal adipose tissue (GAT) were also removed, their weight was measured, and the latter was preserved in formaldehyde 3.7–4.0% buffered to pH = 7 (Panreac, Darmstadt, Germany) for immunohistochemical analysis. The liver was also collected, some portions were preserved in formaldehyde 3.7–4.0% buffered to pH = 7 (Panreac, Darmstadt, Germany) for immunohistochemical analysis and others were stored in TRI Reagent® (Sigma) for RNA extraction.

### 4.4. Isolation of Adipocyte Fraction and Stromal Vascular Fraction Cells

Adipose tissue digestion was done as previously described [8,9]. Briefly, collagenase type II (Sigma-Aldrich) was added to the recovered adipose tissue samples to a final concentration of 1 mg/mL. Samples were incubated in a water bath at 37 °C, for up to 60 min with manual shaking each 10 min. Digested samples were homogenized to single-cell suspensions and passed through a 100 μm cell strainer. After centrifugation at 280× *g* for 10 min at 4 °C, a portion of the supernatant (400 μL for all samples) containing the adipocyte layer was stored in TRI Reagent® (Sigma). The remaining solution was discarded and cells at the bottom, corresponding to the SVF, were resuspended in RPMI-1640 medium supplemented with 10% FBS (Gibco, MA, USA) and 85 units/mL penicillin, 85 μg/mL streptomycin, 62.5 ng/mL of amphotericin B, 10 mM HEPES, and 50 μM 2-mercaptoethanol (all from all from Sigma-Aldrich, St. Louis, USA) and counted in a haemocytometer. Finally, 1 × 10^6^ cells were stored in TRI Reagent® (Sigma).

### 4.5. RNA Isolation and Real-Time PCR Analysis

RNA was extracted from 10^6^ MAT SVF cells of WT and p40^−/−^ mice, as previously described in detail [8,9]. A similar protocol was used for the extraction of RNA from the adipocyte fraction stored in TRI Reagent® (Sigma). Total RNA from 50 to 100 mg of liver was also extracted with TRI Reagent® accordingly to the manufacturer’s instructions. RNA samples were recovered in nuclease-free H_2_O and quantified using Nanodrop ND-1000 apparatus (Thermo Scientific). A maximum of 2.5 μg of RNA was then converted into cDNA using a Maxima^®^ First-Strand cDNA Synthesis kit for RT-quantitative PCR (Fermentas, Thermo Scientific), according to the manufacturer’s instructions. The PCR programme run (25 °C for 10 min; 50 °C for 30 min; 85 °C for 5 min) was performed in a TProfessional Basic Thermocycler (Biometra GmbH, Goettingen, Germany). Real-time PCR was then used for quantification of mRNA expression levels of *Lep*, *Lepr* transcript variant 1, and *Lepr* transcript variant 3 mRNA expression levels with the Kapa SYBR Fast qPCR Kit (Kapa Biosystems Inc., Wilmington, MA) in a Rotor-Gene 6000 (Corbett life science, Sydney, Australia). As reference genes, we used hypoxanthine guanine phosphoribosyl transferase (*Hprt*) and non-POU-domain containing octamer binding protein (*Nono*) as previously described [8,9]. The reaction was performed in a final volume of 10 μL containing 0.2 μM of each specific primer [8,9]: *Nono* forward: GCTCTTTTCTCGGGACGG, *Nono* reverse: GCATTTTTGTACCCTTGACTTGGA; *Hprt* forward: ACATTGTGGCCCTCTGTGTG, *Hprt* reverse: TTATGTCCCCCGTTGACTGA; *Lep* forward: TCAAGCAGTGCCTATCCAGA, *Lep* reverse: AAGCCCAGGAATGAAGTCCA; *Lepr* transcript variant 1 forward: GGGACGATGTTCCAAACCCC, *Lepr* transcript variant 1 reverse: TCTGAAATGGGTTCAGGCTCC; *Lepr* transcript variant 3 forward: TGTCCTACTGCTCGGAACAC, *Lepr* transcript variant 3 reverse: AGAGTGTCCGTTCTCTTTTGGA (all from Tib Molbiol, Berlin, Germany) and 1 × Master Mix plus 1 μL of the newly synthesized cDNA diluted 1/5 (adipocyte fraction) or 1/10 (SVF and liver tissue). The PCR program run was as follows: (1) denaturation at 95 °C, 5 min; (2) amplification in 50 cycles (95 °C, 10 s; 62 °C, 20 s). We analysed real-time PCR data by the comparative threshold cycle (C_T_) method [43]. Individual relative gene expression values were calculated using the following formula: 2 ^− (C^T ^gene of interest − C^T ^constitutive gene)^ [43]. Primers for *Lepr* transcript variant 1 and transcript variant 3 were designed with primer-BLAST tool [44].

### 4.6. Immunohistochemistry and Histopathologic Analysis

The organs/tissues stored in formaldehyde were dehydrated, embedded in paraffin wax, and sections were cut from each block to slides. Some slides were used for immunohistochemistry analysis and one slide was stained with haematoxylin–eosin (HE). For analysis of leptin and *N. caninum* in organs/tissues, a similar protocol to that described in Teixeira et al. [9] was used. Briefly, sections were boiled in a pressure cooker containing citrate buffer (10 mM, pH = 6) for 3 min. For endogenous peroxidase activity blocking, all sections were incubated with 3% hydrogen peroxide in methanol (Merck, Darmstadt, Germany) for 20 min. To eliminate non-specific staining, sections were incubated in a humid chamber for 20 min with normal goat serum (Dako, Glostrup, Denmark) diluted 1:5 in PBS (sections intended for leptin analysis) or normal rabbit serum (Dako, Glostrup, Denmark) diluted 1:5 in PBS with 10% BSA (sections intended for *N. caninum* analysis). Excess serum was then removed, and the sections were incubated overnight, with rabbit anti-mouse leptin polyclonal antibody (Abcam, Cambridge, UK) diluted 1:1000 or only PBS (negative control) for leptin analysis. For *N. caninum* analysis, sections were incubated at room temperature with goat anti-*N. caninum* polyclonal serum (VMRD, Pullman, WA, USA) diluted 1:2000 for 1 h 30 min. Sections were then washed and incubated at room temperature for 30 min with peroxidase-labelled goat anti-rabbit IgG (Abcam) diluted 1:2000 (sections incubated with anti-leptin antibody) or peroxidase-labelled rabbit anti-goat secondary antibody (Millipore, Billerica, MA, USA) diluted 1:1500 (sections incubated with anti-*N. caninum* serum). The colour was developed by incubation with 3,3′-diaminobenzidine (Dako). Sections were then counterstained with Mayer′s haematoxylin (Merck) and a cover glass was added to the slides in the mounting medium Entellan® (Merck). A positive reaction was indicated by the presence of brown staining.

All microphotographs were captured with a ZEISS microscope coupled with a Leica camera, using the Leica Application Suite (LAS), version 4.12.0 software. For leptin quantification in gonadal adipose tissue, 10 to 17 images of sections of adipose tissue from each individual mouse were obtained in a 400× magnification field. As indicated in the acquisition software, each image size was 260 × 195 μm, and therefore had an area of 0.0507 mm^2^. The total area analysed for each mouse was calculated by multiplying the number of photos taken by the area of each photo, that was therefore between 0.5070 and 0.8620 mm^2^. Non-adipocyte cells staining for leptin were manually counted with the aid of the cell counter of ImageJ version 1.45 s to mark each cell counted. In this tissue, whenever it was not possible to distinguish between leptin staining in adipocytes or stromal vascular fraction, the cells were not counted. The total number of counted cells in the group of photos taken from each animal was divided by the total area analysed. For leptin quantification in the liver, on average 38 ± 19 (standard deviation) images of sections of liver from each individual mouse were obtained in a 200× magnification field. As indicated in the acquisition software, each image size was 520 × 390 μm, and therefore had an area of 0.2028 mm^2^. The total number of cells staining with leptin antibody were manually counted with the aid of the cell counter of ImageJ version 1.45 s, in each image. The total number of counted cells was divided by the total area analysed, which was calculated by multiplying the number of photos taken by the area of each photo. The number of cells staining positive for leptin in the liver were analysed on average in a total area of 7.7 ± 3.9 mm^2^ in each individual mouse.

### 4.7. Leptin Measurement in Serum

Leptin in the serum was measured with a mouse leptin ELISA Kit (Merck Millipore, Billerica, MA, USA), following the manufacturer′s instructions.

### 4.8. Statistical Analysis

Statistical significance of the results obtained for *N. caninum*-challenged mice versus the respective controls groups was assessed by the non-parametric Mann–Whitney test, calculated with GraphPad Prism software version 8.3.0. (* *p* ≤ 0.05; ** *p* ≤ 0.01; *** *p* ≤ 0.001; **** *p* ≤ 0.0001). Because the total number of animals in each group is low (n = 6 or 9), non-parametric tests were used [45,46]. Moreover, not all our data followed a normal distribution, as calculated with the normality test Shapiro–Wilk in GraphPad Prism 8.3.0 software.

The results presented are pooled from two or three independent experiments with n = 3 mice/group/experiment, as indicated in respective figure legends. The bars represent median values of each experimental group (the total number of mice analysed per group was six or nine when two or three independent experiments were done, respectively) with each individual mouse being represented by a symbol.

## Supplementary Materials

The following are available online at https://www.mdpi.com/2076-0817/9/7/587/s1, Figure S1: Decreased expression of leptin in the adipose tissue of infected mice, Figure S2: Decreased expression of leptin receptor in the adipose tissue of *Neospora caninum* infected mice, Figure S3: Staining for leptin in the adipose tissue of *Neospora caninum*-infected mice, Figure S4: Detection of *N. caninum* in gonadal adipose tissue and liver of infected wild-type and IL-12/IL-23 p40-deficient C57BL/6 mice, Figure S5: Histopathological analysis of liver sections of *Neospora caninum*-infected mice.
