# Supplementary material for: Modulation of Leptin and Leptin Receptor Expression in Mice Acutely Infected with Neospora caninum
Source: Pathogens. 2020 Jul 17;9(7):587. doi: 10.3390/pathogens9070587 (PMC7399848; doi:10.3390/pathogens9070587)
Supplement: Supplementary file 1 [file pathogens-09-00587-s001.pdf]

pathogens

MDPI

Supplementary Materials

Modulation of Leptin and Leptin Receptor Expression in Mice Acutely Infected with *Neospora caninum*

Luzia Teixeira [1,2,*], Alexandra Correia [1,3,4], Bárbara M. Oliveira [1,2], Ana Pinto [1,2], Paula G. Ferreira [1,2] and Manuel Vilanova [1,3,4]

1 ICBAS—Instituto de Ciências Biomédicas Abel Salazar, Universidade do Porto, Rua de Jorge Viterbo Ferreira, 228, 4050-313 Porto, Portugal; alexandra.correia@ibmc.up.pt (A.C.); bmnoliveira@icbas.up.pt (B.M.O.); arpinto@icbas.up.pt (A.P.); pferreir@icbas.up.pt (P.G.F.); vilanova@icbas.up.pt (M.V.)

2 UMIB—Unidade Multidisciplinar de Investigação Biomédica, Universidade do Porto, Rua de Jorge Viterbo Ferreira, 228, 4050-313 Porto, Portugal

3 i3S-Instituto de Investigação e Inovação em Saúde, Universidade do Porto, Rua Alfredo Allen, 208, 4200-135 Porto, Portugal

4 IBMC—Instituto de Biologia Molecular e Celular, Universidade do Porto, Rua Alfredo Allen, 208, 4200-135 Porto, Portugal

* Correspondence: lmteixeira@icbas.up.pt; (+351) 220428109

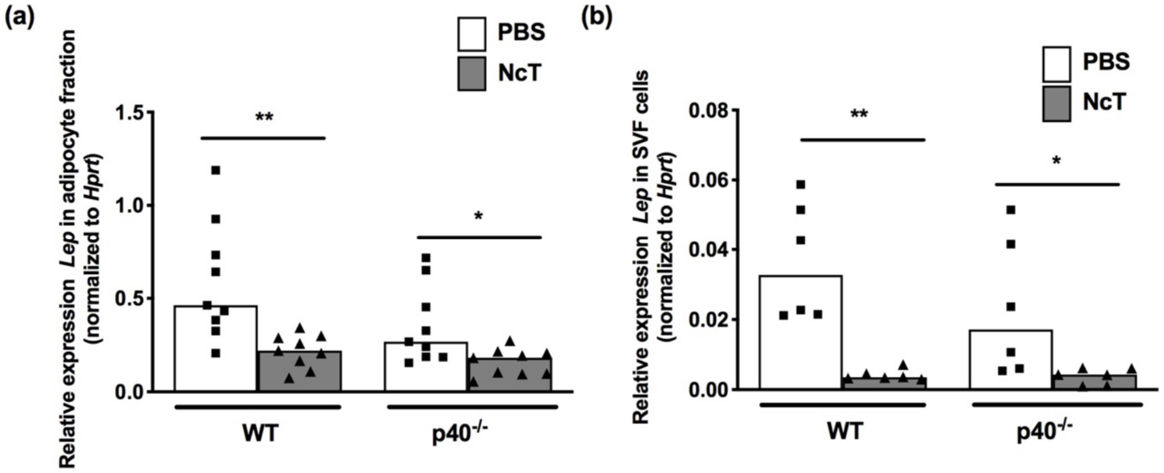

Figure S1. Decreased expression of leptin in the adipose tissue of infected mice. Relative levels of leptin (*Lep*) mRNA normalized to hypoxanthine guanine phosphoribosyl transferase. (*Hprt*) mRNA, detected by real-time PCR in the (**a**) adipocyte fraction or (**b**) stromal vascular fraction (SVF) cells of mesenteric adipose tissue of wild-type (WT) and IL-12/IL-23 p40-deficient (p40−/−) mice 24 hours after intraperitoneal administration of 1 × 10^7 *N.* caninum tachyzoites (NcT) or PBS. Bars represent the median values of (**a**) nine mice or (**b**) six mice per group, with each individual mouse being represented by a symbol. Results are pooled from (**a**) three independent experiments or (**b**) two independent experiments, with three mice per group per experiment. Statistically significant differences between *N.* caninum-challenged and respective control groups are indicated (Mann-Whitney test, *$P < 0.05$; **$P \leq 0.01$).

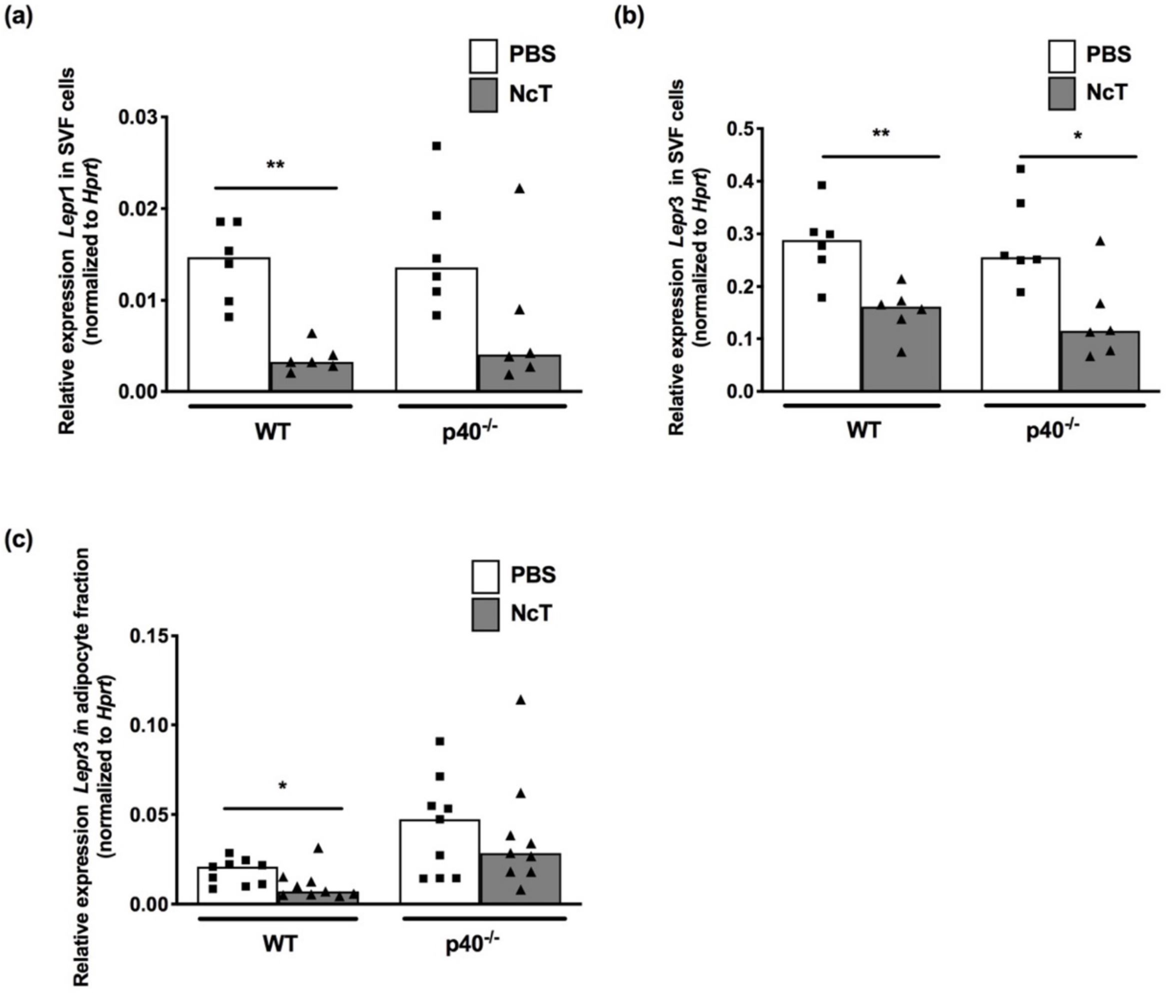

Figure S2. Decreased expression of leptin receptor in the adipose tissue of. *Neospora caninum* infected mice. (**a**) Relative levels of leptin receptor transcript variant 1 (*Lepr*1). mRNA and (**b**) leptin receptor transcript variant 3 (*Lepr*3), mRNA, normalized to hypoxanthine guanine. phosphoribosyl transferase (*Hprt*) mRNA, detected by real-time PCR in stromal vascular fraction. (SVF) cells of mesenteric adipose tissue of wild-type (WT) and IL-12/IL-23 p40-deficient (p40-/-) mice. 24 h after intraperitoneal administration of 1 × 10^7 *N. caninum* tachyzoites (NcT) or PBS, as indicated. Bars represent the median values of six mice per group, with each individual mouse being. represented by a symbol. These are pooled results from two independent experiments with three. mice per group per experiment. (**c**) Relative levels of leptin receptor transcript variant 3 (*Lepr*3). mRNA, normalized to hypoxanthine guanine phosphoribosyl transferase (*Hprt*) mRNA, detected by. real-time PCR in adipocyte fraction of mesenteric adipose tissue of wild-type (WT) and IL-12/IL-23. p40-deficient (p40−/−) mice 24 hours after intraperitoneal administration of 1 × 10^7 *N. caninum.* tachyzoites (NcT) or PBS, as indicated. Bars represent the median values of nine mice per group, with each individual mouse being represented by a symbol. These are pooled results from three. independent experiments with three mice per group per experiment. Statistically significant. differences between *N. caninum*-challenged and respective control groups are indicated (Mann-Whitney test, *$P < 0.05$; **$P \leq 0.01$).

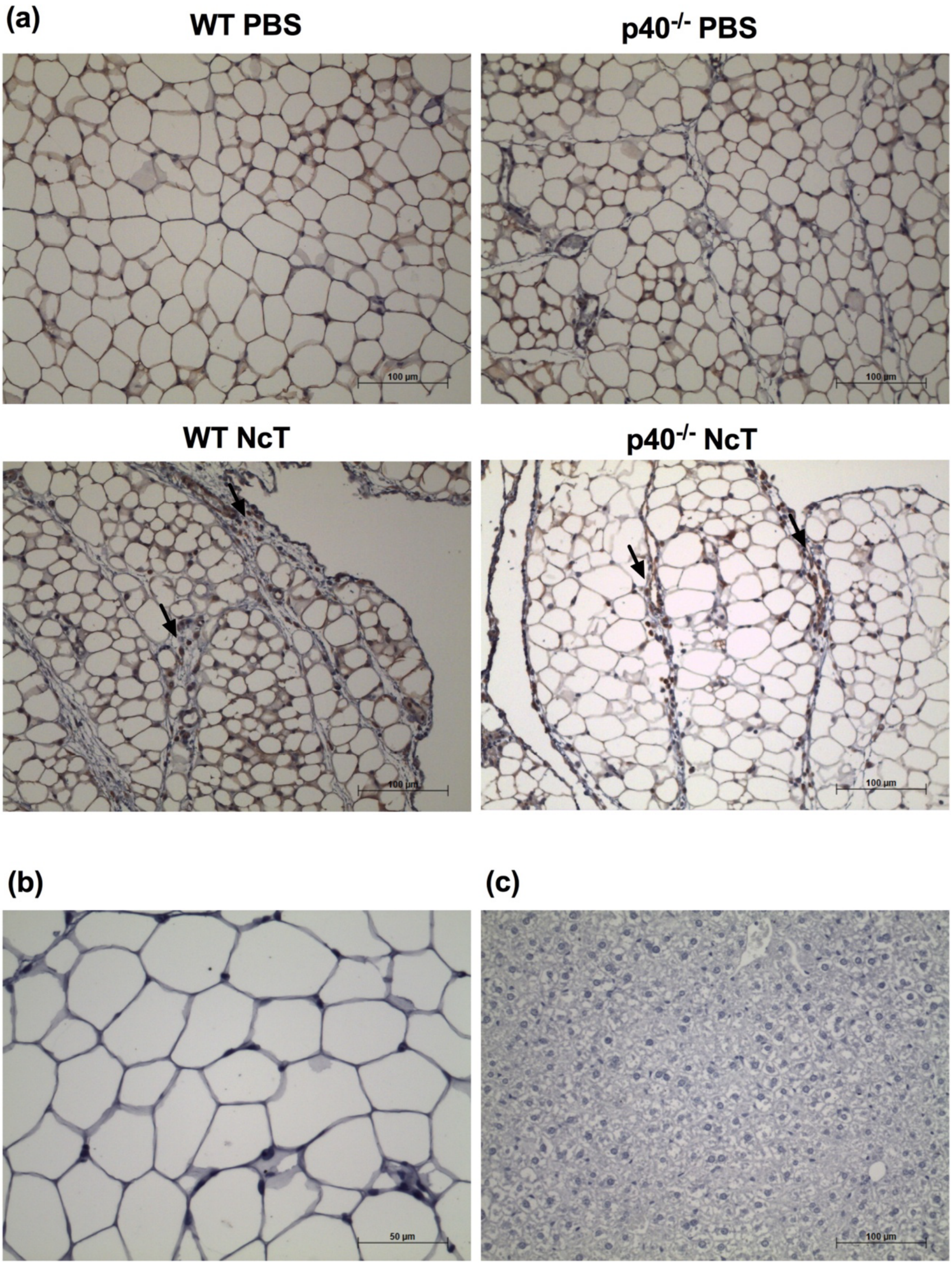

Figure S3. Staining for leptin in the adipose tissue of *Neospora caninum*infected. mice. **(a)** Representative images of leptin detected by immunohistochemistry in gonadal adipose tissue of wild-type (WT) and IL-12/IL-23 p40-deficient (p40−/−) mice 24h after intraperitoneal administration of 1 × 10^7 *N. caninum* tachyzoites (NcT) or PBS. Adipose tissue was specifically stained (brown coloration) with a polyclonal anti-mouse leptin antibody and counterstained with haematoxylin. Bar = 100 μm. These are illustrative examples of two independent experiments with n = 3 per group. Sections of (**b)** adipose tissue or **(c)** liver incubated with PBS instead of the polyclonal anti-mouse leptin antibody (negative control). Bar = 50 μm in **(b)** or 100 μm in **(c)**.

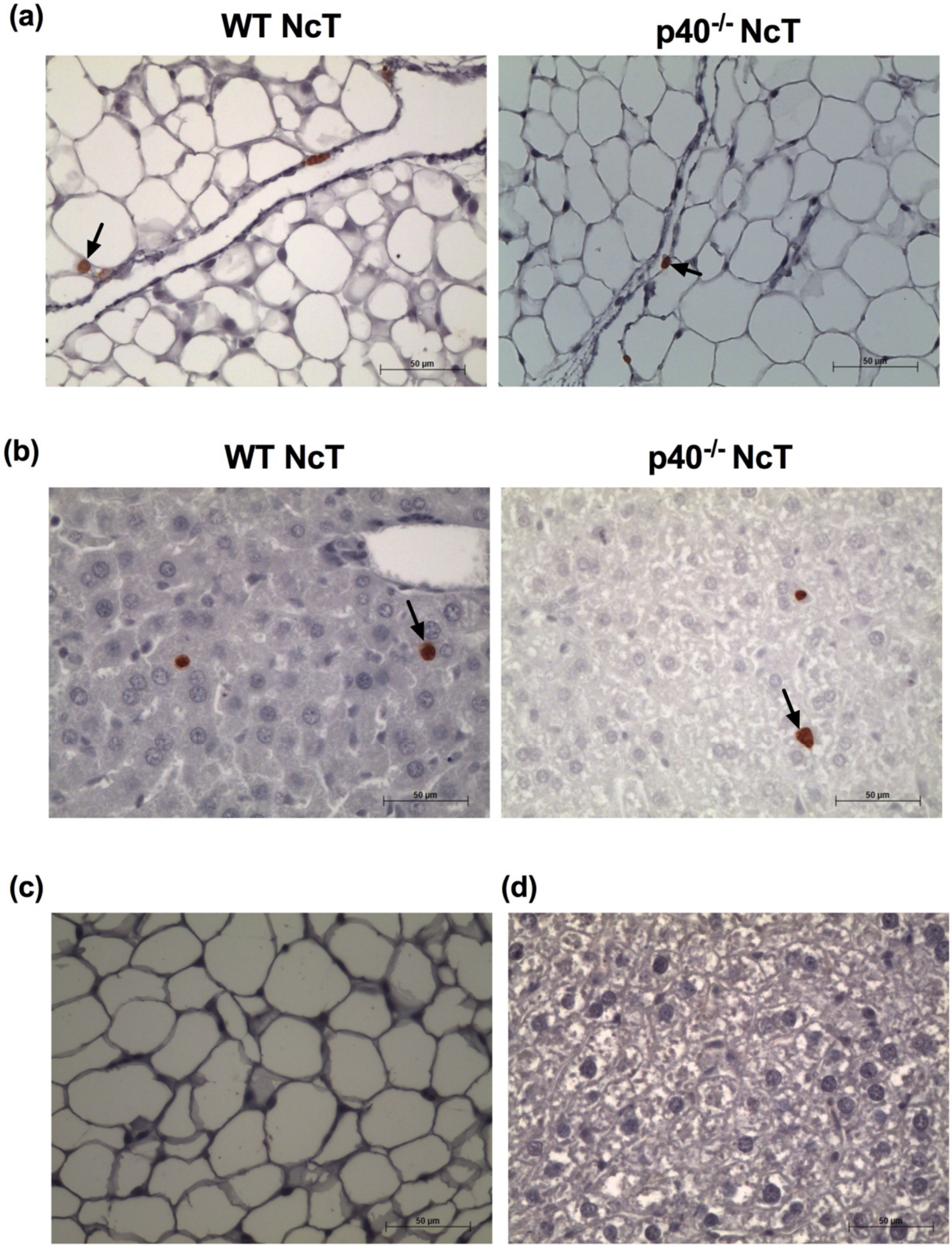

Figure S4. Detection of *N. caninum* in gonadal adipose tissue and liver of. infected wild-type and IL-12/IL-23 p40-deficient C57BL/6 mice. Representative images showing. parasitic forms in (**a**) gonadal adipose tissue and (**b**) liver of wild-type (WT) or IL-12/IL-23 p40-deficient (p40−/−) C57BL/6 mice 24h after administration of 1 × 10^7 N. caninum tachyzoites (NcT), detected by immunohistochemistry. Sections of adipose tissue and liver were specifically stained (brown coloration, indicated by arrows) with a polyclonal anti-*N. caninum* serum and counterstained with haematoxylin. This is one representative result of two independent experiments with three mice per group per experiment. Sections of (**c**) adipose tissue or (**d**) liver of a WT non-infected mice (negative control). Bar = 50 µm in all micrographs.

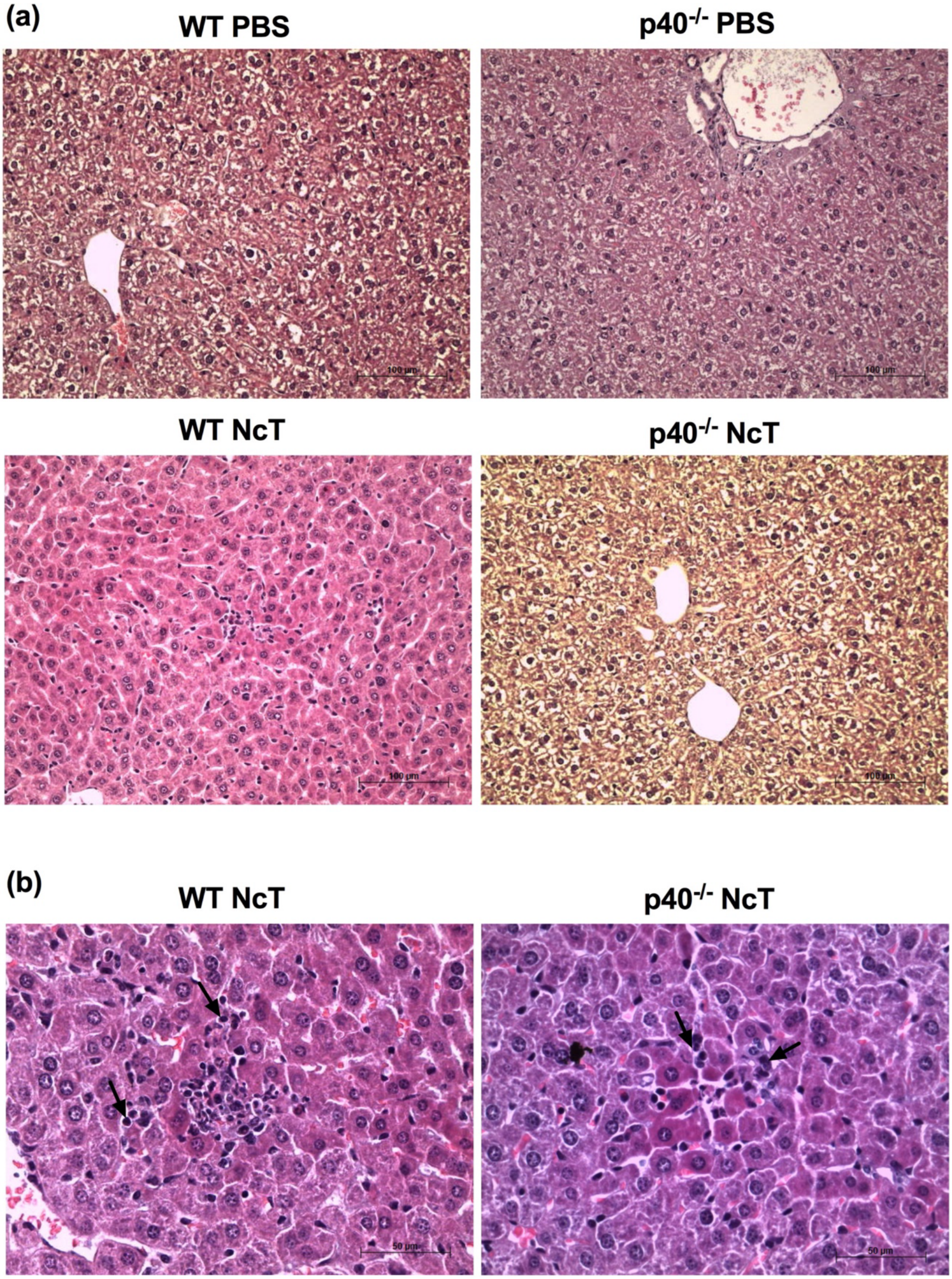

Figure S5. Histopathological analysis of liver sections of *Neospora caninum*infected mice. Micrographs of haematoxylin-eosin-stained liver sections of wild-type (WT) and IL- 12/IL-23 p40-deficient (p40−/−) C57BL/6 mice 24h after intraperitoneal administration of 1 × 10^7 *N. caninum* tachyzoites (NcT) or PBS, as indicated. (**a**) Cellular infiltrates are evident in the liver of infected WT mice. In infected p40−/− mice cellular infiltrates are more difficult to observe. (**b**) Higher magnification of small foci of hepatic necrosis rarely seen in infected WT and scarcely observed in p40-/- infected mice. Inflammatory cells were observed not only in necrosis foci but also inside the 7

sinusoids (arrows). This is one representative result of two independent experiments with three mice per group per experiment.
